# Substantial population structure of *Plasmodium vivax* in Thailand facilitates identification of the sources of residual transmission

**DOI:** 10.1371/journal.pntd.0005930

**Published:** 2017-10-16

**Authors:** Veerayuth Kittichai, Cristian Koepfli, Wang Nguitragool, Jetsumon Sattabongkot, Liwang Cui

**Affiliations:** 1 Mahidol Vivax Research Unit, Faculty of Tropical Medicine, Mahidol University, Bangkok, Thailand; 2 The Walter and Eliza Hall Institute of Medical Research, Parkville, Victoria, Australia; 3 Department of Molecular Tropical Medicine, Faculty of Tropical Medicine, Mahidol University, Bangkok, Thailand; 4 Department of Entomology, Pennsylvania State University, University Park, Pennsylvania, United States of America; University of Florida, UNITED STATES

## Abstract

**Background:**

*Plasmodium vivax* transmission in Thailand has been substantially reduced over the past 10 years, yet it remains highly endemic along international borders. Understanding the genetic relationship of residual parasite populations can help track the origins of the parasites that are reintroduced into malaria-free regions within the country.

**Methodology/Results:**

A total of 127 *P*. *vivax* isolates were genotyped from two western provinces (Tak and Kanchanaburi) and one eastern province (Ubon Ratchathani) of Thailand using 10 microsatellite markers. Genetic diversity was high, but recent clonal expansion was detected in all three provinces. Substantial population structure and genetic differentiation of parasites among provinces suggest limited gene flow among these sites. There was no haplotype sharing among the three sites, and a reduced panel of four microsatellite markers was sufficient to assign the parasites to their provincial origins.

**Conclusion/Significance:**

Significant parasite genetic differentiation between provinces shows successful interruption of parasite spread within Thailand, but high diversity along international borders implies a substantial parasite population size in these regions. The provincial origin of *P*. *vivax* cases can be reliably determined by genotyping four microsatellite markers, which should be useful for monitoring parasite reintroduction after malaria elimination.

## Author summary

This study presents an updated view of the *P*. *vivax* populations along the Thai-Myanmar and the Thai-Cambodian borders. Genotyping of parasite samples collected after intensified malaria control demonstrated that despite the decline in overall transmission intensity, the genetic diversity of the *P*. *vivax* parasites remained high. Parasite populations from three border provinces showed clear genetic separation. This indicates successful interruption of parasite gene flow within Thailand, but suggests frequent parasite migration across international borders. From the analysis of 10 microsatellite markers, we further refined a set of four that are sufficient for distinguishing the provincial origins of these parasites, which should allow tracking of parasite introduction among these provinces.

## Introduction

Although the global incidence of malaria has been greatly reduced in recent years, *Plasmodium vivax* remains the most geographically widespread human malaria parasite [1]. In Thailand, intensified malaria control has resulted in ~50% reduction of clinical cases over the past 10 years. In parallel, the distribution of malaria cases in Thailand has become more heterogeneous. The central region is virtually malaria free, whereas malaria remains endemic along the western border with Myanmar, the northeastern border with Laos, the eastern border with Cambodia, and to a lesser extent, the southern border with Malaysia [2]. As a result, malaria along these four international borders now accounts for 95% (56, 17, 13 and 9%) of the total malaria cases in Thailand [3]. An increasing proportion of the remaining infections is caused by *P*. *vivax*, which is now the source of 63% of all clinical cases [3]. In 2015, *P*. *vivax* annual parasite incidence was 0.37 per 1000. Given the successful interruption of transmission in other parts of the country, information on diversity and genetic relationship of parasites from the remaining transmission hotspots can help target control programs to track the sources of remaining infections and to direct control programs towards remaining transmission hotspots. This is particularly relevant for *P*. *vivax*, since the dormant liver hypnozoites can relapse months or even years after the initial infection in seemingly parasite-free migrants.

Genotyping of microsatellite (MS) markers [4,5], polymorphic antigens [6], mitochondrial DNA [7,8], and genome-wide single nucleotide polymorphisms (SNPs) [9] has revealed high levels of diversity among *P*. *vivax* populations on both global and local scales. Whole-genome sequencing of >400 isolates has confirmed high *P*. *vivax* diversity and population structure following continental origins of the isolates [10], as well as a clear separation of parasite populations between western Thailand and Cambodia [11]. Earlier studies on *P*. *vivax* diversity in Thailand in the 1990s and early 2000s, when transmission levels were significantly higher, found high diversity of several antigens and population genetic differentiation between regions [12,13]. More recently, genotyping of different polymorphic markers further confirmed the high diversity in most regions [14–16]. However, a recent study with the *msp3β* gene revealed contrasting genetic structure of the *P*. *vivax* populations between the southern and northwestern border regions, with the northwestern parasite population showing very high genetic diversity as compared to southern population with the same *msp3β* allele [17]. To further characterize the population genetic structure and relatedness of *P*. *vivax* populations in Thailand after the scaling-up of malaria control in recent years, we have genotyped *P*. *vivax* parasite samples from three residual malaria transmission foci along the western and eastern borders.

## Results

### Genetic diversity

**Fig 1:**
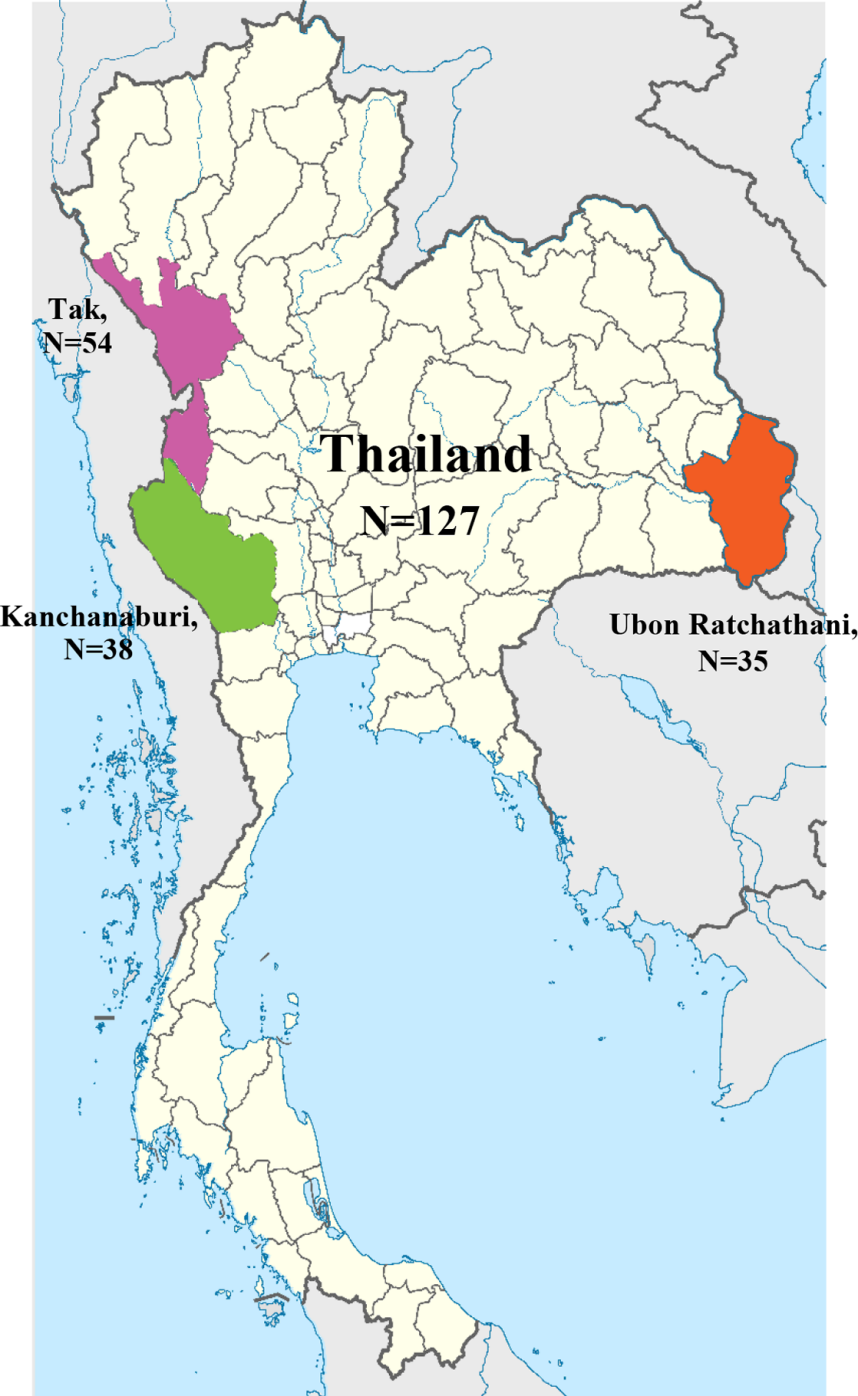
Map showing three provinces in Thailand where samples were collected (modified from https://commons.wikimedia.org/wiki/Atlas_of_Thailand).

A total of 127 *P*. *vivax* isolates from two western border provinces and one eastern border province of Thailand (**Fig 1**) were genotyped using 10 MS markers [18]. Complete genotyping data for all 10 loci was obtained for 112 isolates (87.5%). Three samples with fewer than five MS markers genotyped were removed from analysis. The predominant allele of each locus in each sample was used for population genetic analysis. MS2 was the most diverse marker with >17 alleles in each parasite population, whereas MS1 was the least diverse with only 6–7 alleles (**S1 Fig** and **S1 Table**). For all markers except MS5, at least one allele was shared among the three populations; the most extensive allele sharing was found with MS1 (3 alleles) and MS6 (4 alleles). Parasite diversity was high; when all sites were combined, the mean expected heterozygosity (*H*_*E*_) was 0.851, and it was not significantly different among the three sites (0.837, 0.850, and 0.867 for Kanchanaburi, Ubon Ratchathani and Tak, respectively) (**Table 1**).

**Table 1:** The mean number of alleles, the multiplicity of infection and the expected heterozygosity (*H*_*E*_) per locus.

Provincial populations	N	Mean *H*_*E*_	Mean allele numbers*	Mean allelic richness^a^	MOI^#^ (10 MS)	MOI (2 MS)	% multi clones
**Tak**	54	0.867	13.7	12.806	1.358	1.222	23.7
**Kanchanaburi**	37	0.837	10.6	10.565	1.132	1.132	14.6
**Ubon Ratchathani**	33	0.850	10.2	10.199	1.258	1.258	19.7
**Total**	**124**	**0.851**^**ns**^	**28.3**	**28.395**^**ns**^^**,**^ ^**b**^	**1.249**	**1.205**	**19.3**^**f**^

* Significant by Mann-Whitney U test of no. allele between Ubon Ratchathani and Tak.

^#^ Significant by Mann-Whitney U test of MOI between Ubon Ratchathani and Kanchanaburi and between Kanchanaburi and Tak. 10 MS and 2 MS indicate MOI as the highest number of observed alleles at any of the 10 loci and at any of the two most diverse loci, respectively.

^a^ Allelic richness based on a minimum sample size of 32 haploid individual samples.

^b^ Allelic richness based on a minimum sample size of 120 haploid individual samples.

^ns^ Not significant among sites by Mann-Whitney U test and Kruskal Wallis test.

^f^ Significant difference in the percentage of multiclonal infections among the three sites (p < 0.05, Pearson Chi-Square test).

Twenty four (19.3%) parasite isolates contained multiclonal infections (more than one peak for at least one MS marker), which resulted in a mean multiplicity of infection (MOI) of 1.297 (**Table 1**). Tak had a higher proportion of multiclonal infections (23.70%) than Kanchanaburi (14.60%) and Ubon Ratchathani (19.70%); the differences were significant among all sites with pairwise comparisons (*p* < 0.05, Pearson Chi-square Test) (**Table 1**).

Despite the hypoendemic nature of malaria in Thailand, the haplotype diversity was extremely high. A total of 116 haplotypes were observed and no haplotypes were shared among the three sites. In Tak, identical haplotypes 25, 79, 92, and 109 were shared by two, three, and four parasites, respectively, whereas in Kanchanaburi, two parasite isolates carried the same haplotype (**S2 Table**).

### Genetic differentiation between populations

**Fig 2:**
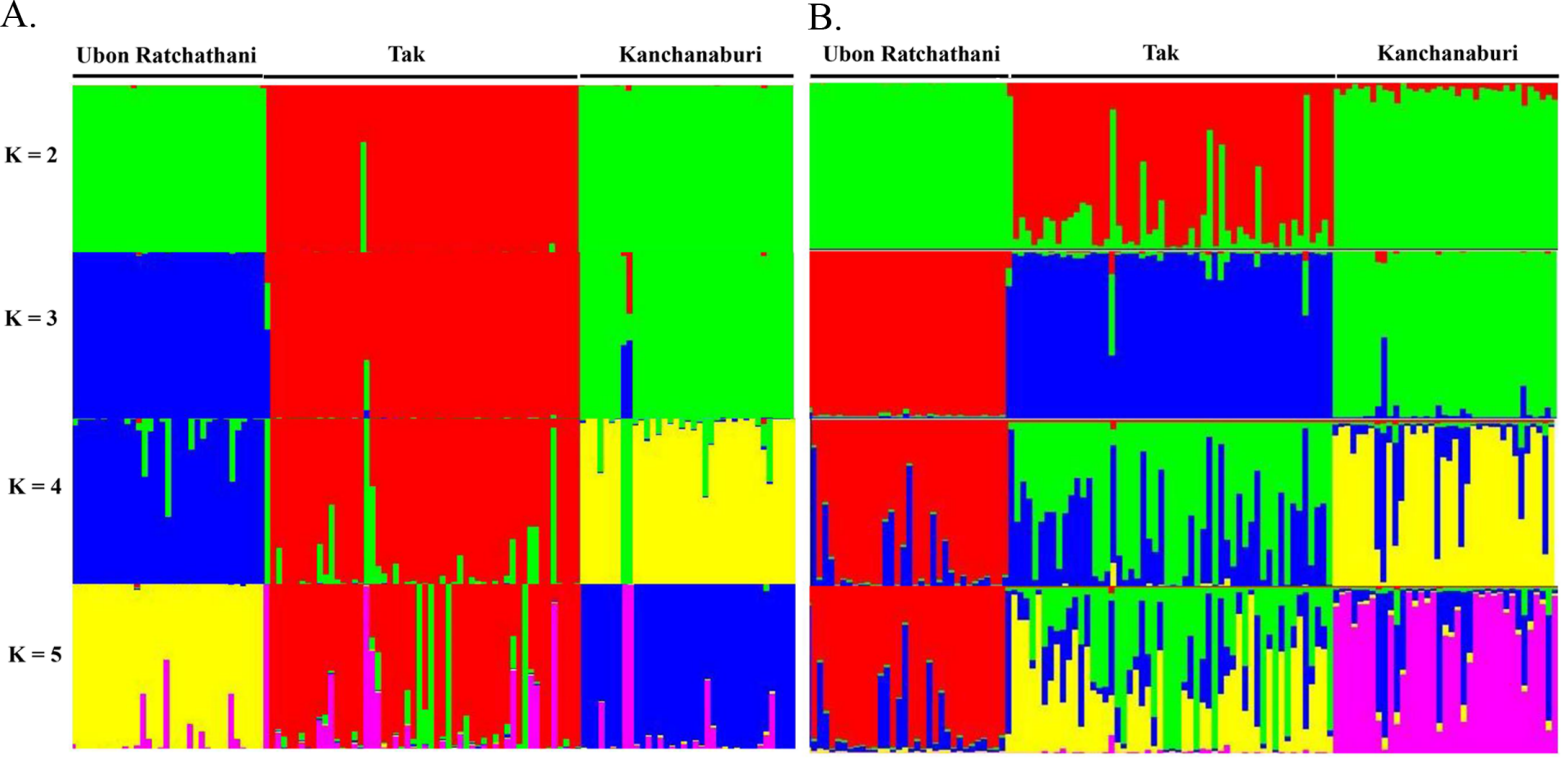
Population genetic structure of *P*. *vivax* in three provinces (K = 2–5). The structure was plotted by using 10 (A) and (B) 4 MS markers (MS2, MS6, MS10 and MS12).

Significant genetic differentiation was observed among the three parasite populations (*Fst* = 0.113–0.126, *p* < 0.05, **Table 2**). STRUCTURE analysis showed a clear distribution pattern of parasite haplotypes. When K = 3, isolates clustered according to their provincial origin (**Fig 2A**).

**Table 2:** Genetic differentiation (*Fst*) of *P*. *vivax* populations.

Provinces	Tak	Kanchanaburi
**Kanchanaburi**	0.1134**	
**Ubon Ratchathani**	0.1255**	0.1154**

P values obtained after permutation test.

**; p < 0.01. Genetic difference by province from multiple comparisons: p < 0.0167.

**Fig 3:**
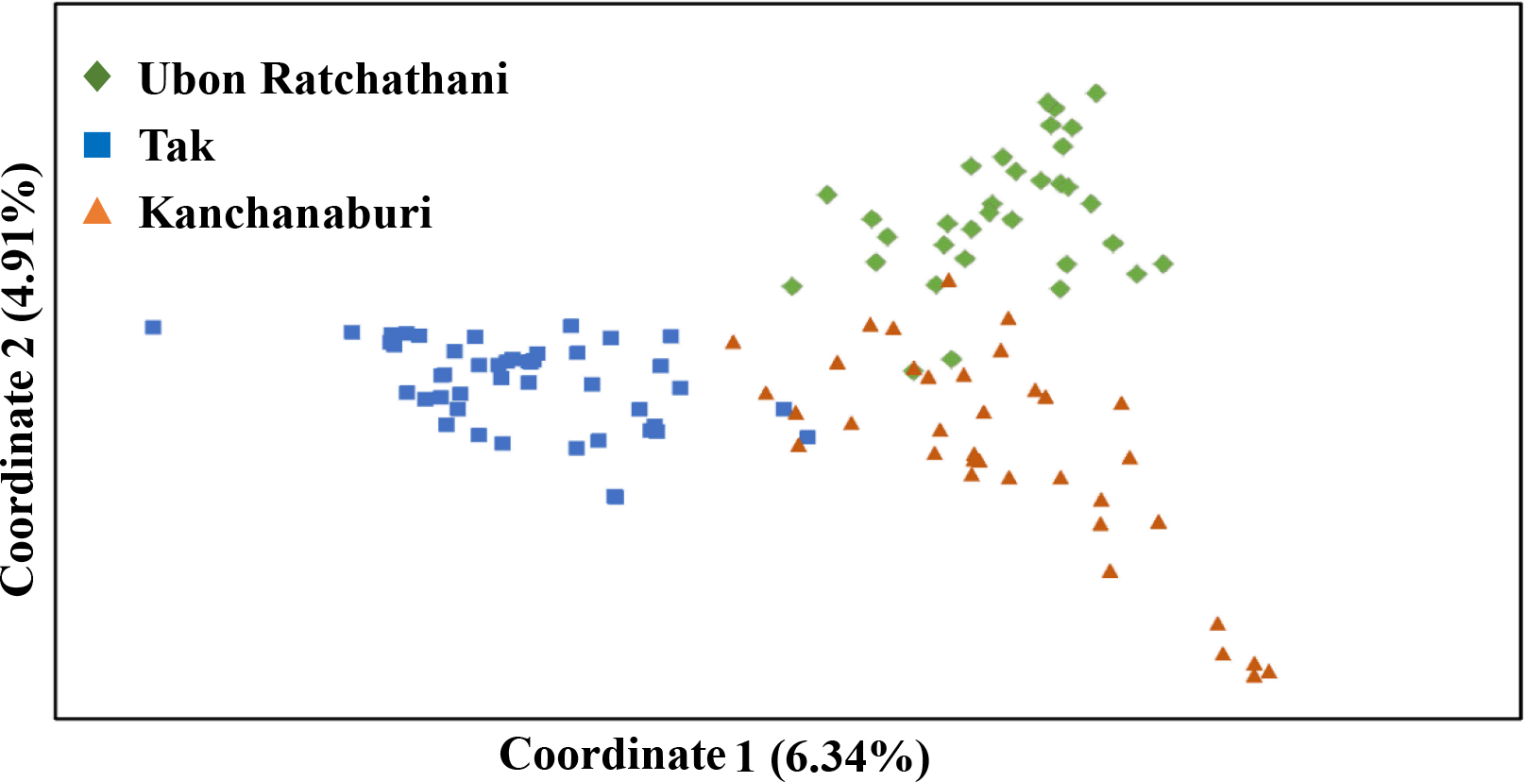
Principal coordinate analysis of *P*. *vivax* haplotypes from three provinces.

Principal coordinate analysis (PCoA) confirmed the genetic separation of the three parasite populations. In line with the STRUCTURE analysis, PC1 separated Tak from Kanchanaburi and Ubon Ratchathani, while PC2 separated Tak from Kanchanburi (**Fig 3**). PC1 and PC2 explained 6.34% and 4.91% of the variance in the data, respectively. Nevertheless, parasites collected from each province had a few haplotypes that shared their phylogenetic relationship. Weak but significant correlation was found between genetic and geographic distances (Mantel rank test, r^2^ = 0.1993, *p* < 0.05) (**S2 Fig**).

**Fig 4:**
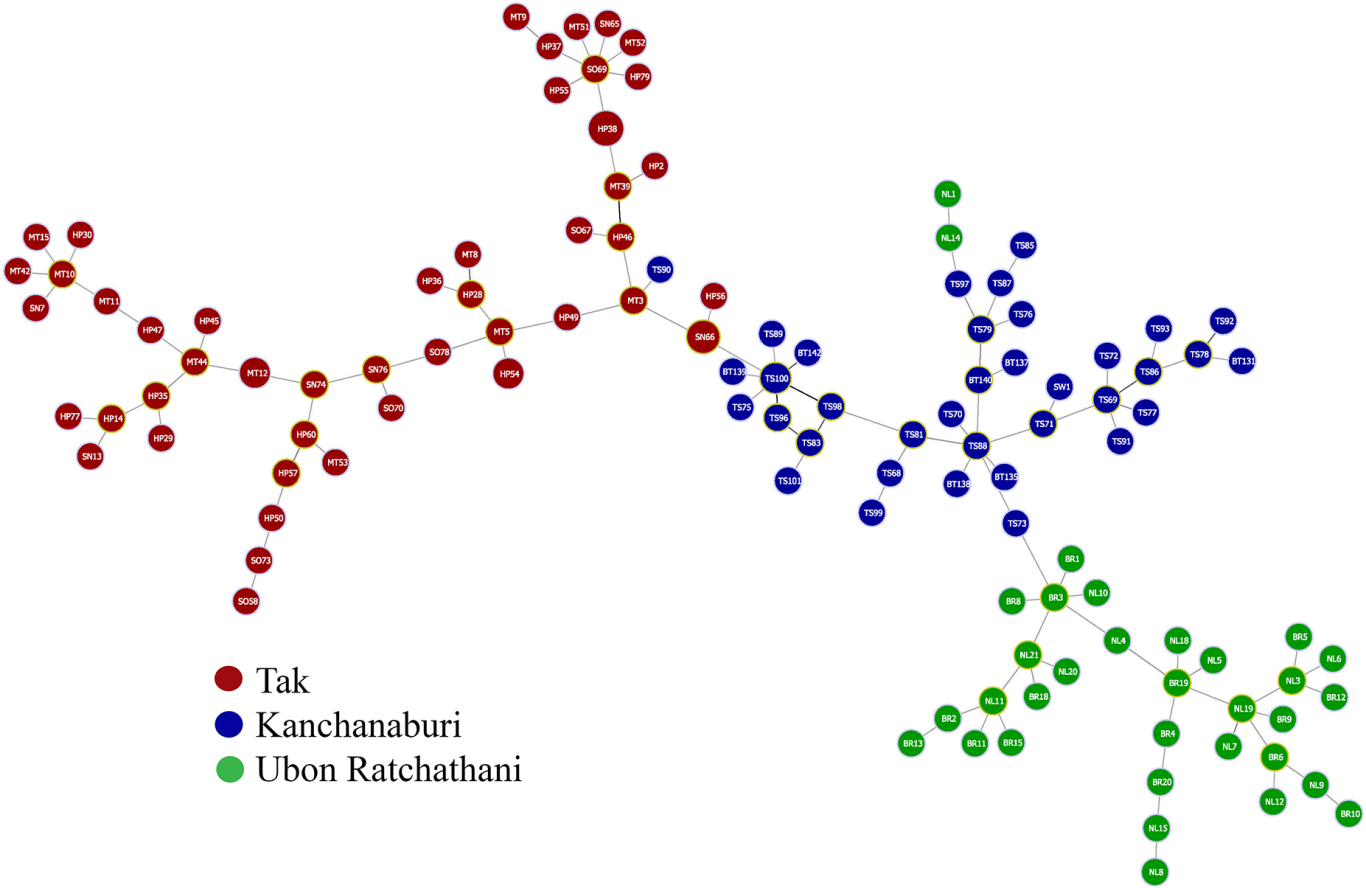
Minimum spanning tree of parasite genotypes constructed using the goeBURST algorithm. Each circle represents a haplotype. Colors indicate the different provinces where the parasites were collected. Sizes of the circles correspond to the numbers of parasites within each haplotype.

The separation of these three parasite populations was further demonstrated by the minimum spanning tree analysis, which revealed a similar pattern of relatedness of parasite isolates by provinces (**Fig 4**). Only a few haplotypes clustered with parasites from other provinces.

### Parasite population size changes

Effective population size (*N*_*E*_) was large in all provinces (**Table 3**). The overall *N*_*E*_ was estimated to be 8704 [95% CI: 3741–19828] using the infinite allele model (IAM), while it was 3–4 fold larger based on the stepwise mutational model (SMM). Significant linkage disequilibrium (LD), suggesting of inbreeding, was observed in Tak (*I*^*S*^_*A*_ = 0.0679, *p* < 0.00001) and Kanchanaburi (*I*^*S*^_*A*_ = 0.1245, *p* < 0.00001), whereas LD did not reach significance in Ubon Ratchathani (*I*^*S*^_*A*_ = 0.0109, *p* = 0.0769).

**Table 3:** Linkage disequilibrium and effective population size of the three *P*. *vivax* populations.

Provincial populations	Linkage disequilibrium*		Effective Population Sizes (*N*_*e*_)			
	*I*^*S*^_*A*_	*P-value*	*SMM*	95% CI	*IAM*	95% CI
**Tak**	0.0679	< 1.00 x 10^−5^	43724	18790–99601	10259	4408–23368
**Kanchanaburi**	0.1245	< 1.00 x 10^−5^	28913	12425–65861	8092	3477–18432
**Ubon Ratchathani**	0.0109	7.69 x 10^−2^	34015	14617–77484	8889	3820–20248
Total	0.1092	< 1.00 x 10^−5^	32797	14094–74710	8704	3741–19828

* Recombination rate (*μ*) of *P*. *falciparum* of 1.59 x 10^−4^ (95% confidence interval: 6.98 x 10^−5^, 3.7 x 10^−4^) was used. *I*^*S*^_*A*_, standardized index of association; SMM, stepwise mutational model; IAM, infinite allele model.

To detect whether there were significant changes in the parasite population sizes, we performed BOTTLENECK analysis using SMM (**Table 4**). The models detected significant deficiency in *H*_*E*_ from the mutation-drift equilibrium, indicating events of population size reduction with possible clonal expansion in all areas.

**Table 4:** Bottleneck analysis ^#^.

*Provincial populations*	SMM		
	Excess-*H*_*E*_	Deficient-*H*_*E*_	2-tails
Tak	0.995	*0*.00684*	*0*.0137*
Kanchanaburi	0.999	*0*.00146*	*0*.0029*
Ubon Ratchathani	0.997	*0*.00488*	*0*.0098*

^#^ To test deviation from mutational drift equilibrium, data were analyzed under the SMM. Excess-*H*_*E*_; *p*-value for excess of *H*_*E*_ under one-tailed analysis. Deficient-*H*_*E*_; *p*-value for deficiency of *H*_*E*_ under one-tailed analysis.

* Statistically significant at *p* < 0.05.

### Minimum number of MS markers for differentiating the three parasite populations

**Fig 5:**
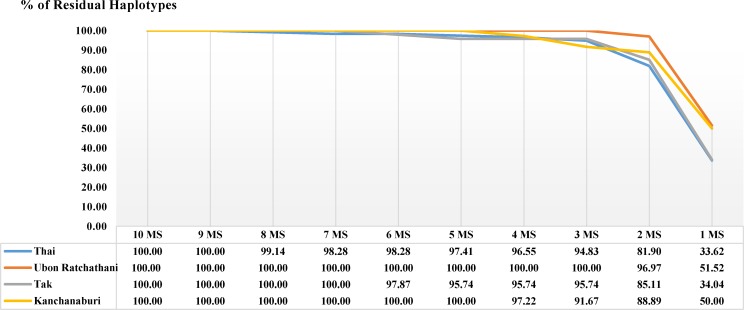
Optimal panel of MS markers and determination of haplotype loss during the removal of each MS. The removal of MS was prioritized from lower to higher *H*_*E*_ according to the X-axis. The remaining haplotypes after removal were shown by the percentages of all haplotypes.

We sought to identify a minimum set of markers suited to differentiate parasite populations based on their provinces of origin, and for differentiating parasites within each province. Stepwise removal of MS markers based on their *H*_*E*_ ranking, starting with the least diverse, showed that four highly diverse MS markers (MS2, MS6, MS12 and MS20) were enough to distinguish more than 96% of all haplotypes (112/116 haplotypes) in the study populations (**Fig 5**and **S3 Table**), and >94% in each province. Due to subtle differences in the diversity of the MS markers in different provinces, the optimal set of haplotypes for each province differed slightly (**S3 Table**). A similar cluster pattern was obtained when performing STRUCURE analysis using these four MS markers as compared with that using all 10 MS markers (**Fig 2B**).

## Discussion

This study presents an updated analysis on *P*. *vivax* diversity and population structure along the Thai-Myanmar and the Thai-Cambodian borders with samples collected after malaria control had been intensified and transmission reduced. Our results clearly show that despite reduction of malaria prevalence in recent years, *P*. *vivax* diversity (*H*_*E*_) remained high. There were still ~20% of the isolates containing mixed-strain infections, and MOI was only slightly lower than in studies conducted 15–20 years ago in Thailand [5,12]. Similarly high *P*. *vivax* diversity despite low prevalence has been reported from other countries [19–21]. Compared to a previous study [4] which used a nearly identical set of microsatellite markers to study isolates from around the world, our observed *H*_E_ (0.84–0.87) in Thailand are similar to those in Cambodia (0.87) and Vietnam (0.84) and clearly higher than those in hypoendemic regions including Peru (0.71), Brazil (0.74), and central Asia (0.75). Thus *H*_E_ generally tracks transmission intensity [4,22]. Interestingly, despite the higher transmission intensity in the southwest Pacific, *H*_E_ in Solomon Islands (0.81) and Papua New Guinea (0.80–0.82) are similar to those in Thailand, Cambodia, and Vietnam, if not lower. This may reflect the more restricted gene flow imposed by the island geography of these areas compared to the mainland Southeast Asia. The maintenance of high genetic diversity in low-transmission areas of Southeast Asia may be due to a large proportion of asymptomatic and submicroscopic infections [23,24]. In addition, relapses from hypnozoites may also be frequent [25]. Both processes favor the maintenance of genetic diversity.

The highly diverse *P*. *vivax* populations from the three Thai provinces are in sharp contrast to data from southern Thailand, where sequencing of the *Pvmsp3β* gene revealed the same genotype in all of 28 isolates [17]. Even though microsatellites have a higher mutation rate than antigens, the results based on *Pvmsp3β* suggest that *P*. *vivax* population in southern Thailand is clonal or near-clonal. Though truly clonal expansion–i.e. many isolates sharing the same haplotype–has been reported only sporadically from other regions, e.g., Uzbekistan and Ethiopia [4,26], it may be a good indicator of that the parasite population size has been reduced to very low levels. On the other hand, our results suggested that parasite population sizes in our study sites were still large and this may represent the general situation in the Greater Mekong Subregion (GMS). Therefore, more intensified control efforts are needed to bring down the relatively large parasite populations during the malaria elimination phase in this region.

As control efforts intensify with the aim of malaria elimination in the GMS by 2030, the remaining parasite transmission foci are expected to be localized along international borders and separated by areas with extremely low or no malaria transmission. Such geographical separation eventually will result in parasite population division. The Mantel test detected a weak correlation between the genetic distance and the geographic distance among our sites (S2 Fig). However, given the small number of populations tested, this correlation may be coincidental. It is interesting to note that the east-west differentiation between Kanchanaburi and Ubon Ratchathani is less strong than between the Kanchanaburi and Tak, as revealed by the structure analysis at K = 2. This could be due to the mountainous terrains on the western border, which limit the gene flow between the two western sites.

Another factor that may have an impact on parasite population division between different sites is malaria vectors. *P*. *falciparum* has been shown to be adapted to local vectors, e.g. infectivity of Asian isolates is lower in African vectors than in local vectors [27]. Furthermore, *P*. *vivax* populations in Mexico have different infectivity in two local *Anopheles* species, and parasite population structure was associated with vector distribution [28,29]. Analogously, we can speculate that the highly divergent vector systems in the GMS may be partially responsible for the parasite genetic structures. Different distribution patterns of malaria vectors between western and eastern regions of Thailand were reported [30]. Major vectors in western Thailand are *An*. *dirus*, *An*. *minimus* and *An*. *maculatus* complexes, whereas *An*. *barbirostris* is distributed in Ubon Ratchathani. The latter species has been shown to be a vector for *P*. *vivax* [31,32] and may explain differentiation of parasites in Ubon Ratchathani from the western provinces. Likewise, *An*. *harisoni* is restricted to Kanchanaburi [33] and may contribute to parasite differentiation in this area. It remains to be determined whether these vector species have differential capacity for different *P*. *vivax* genotypes.

Once malaria transmission becomes very low, the identification of the source of residual infections and potential reintroduction becomes increasingly important [34]. The use of appropriate molecular markers that can clearly separate parasite populations will allow us to identify the sources of parasite introduction. This is especially relevant if parasites are introduced to regions where malaria has been eliminated. As the parasite population evolves over time, the choice of suitable markers will need to be updated and tailored to the questions at hand. For Thailand, genotyping the parasites can be particularly useful if there are malaria resurgence in malaria-free regions of the country. On the other hand, distinguishing local transmission from cross-border reintroduction in areas along the national border may still be challenging. In these areas, local parasite populations likely do not display a clear genetic structure due to heavy cross-border human traffic. Further studies using parasites from both sides of the border will be required.

Our findings that a small panel of MS markers can allocate haplotypes to their provincial origin provide a proof of principle as well as important baseline information for the malaria control programs. Future detection of local cases in areas that are assumed to be malaria-free would indicate that parasite reservoirs are overlooked and that intensified control activities should be implemented. In contrast, detection of cases acquired from border regions would call for heightened efforts for malaria control among migrants.

## Materials and methods

### Ethics statement

Written informed consent was obtained from all blood donors or their legal guardians for participants under the age of 18. This study was approved by the Institutional Review Boards of Mahidol University and the Pennsylvania State University.

### Study sites

Malaria transmission in Thailand is perennial following the patterns of rainfall, and *P*. *vivax* transmission typically has two peaks: one in July–September and one in November [12]. Parasite samples were collected from Tak and Kanchanaburi provinces in western Thailand bordering Myanmar and Ubon Ratchathani Province in eastern Thailand bordering Cambodia and Laos (**Fig 1**). The western provinces are the traditionally high malaria transmission areas. In 2016, however, Ubon Ratchathani reported the highest number of malaria cases in Thailand, whereas Tak and Kanchanaburi were the second and fourth highest, respectively [3]. Malaria in Thailand has decreased significantly in recent years due to intensified national control. According to the annual malaria report (http://203.157.41.215/malariar10/index_newversion.php) by the Thailand Malaria Elimination Program, Ministry of Public Health, the number of malaria cases in Kanchanaburi has continually declined since 2012, with the annual number of 4122 in 2012, 2623 in 2013, 1739 in 2014, 1081 in 2015, to 539 cases in 2016. A similar decline was observed in Tak, with the annual case numbers of 13706, 1279, 6306, 3259 and 1364 from 2012–2016. In contrast, Ubon Ratchathani saw a rise in the malaria cases from 1119 cases in 2012, to 1248 cases in 2013, and to 8834 in 2014 after which a decline was seen to 3436 in 2015 and 788 cases in 2016.

### Parasite sample collection

Blood samples were collected from symptomatic patients attending local malaria clinics. Diagnosis was based on microscopy of Giemsa-stained thin and thick smears and ~100 *μ*l of finger-prick blood from *P*. *vivax* patients were spotted on filter paper, dried and stored in individually zipped plastic bags before DNA extraction. A total of 127 *P*. *vivax* samples were collected, including 38 collected in 2013–2014 from patients attending the malaria clinics in Sai Yok and Srisawat districts, Kanchanaburi, 54 samples in 2013–2015 in Tha Song Yang District, Tak Province, and 35 samples in 2015–2016 in Bun Tharik and Na Chaluai districts, Ubon Ratchathani. The distance between the sites in the two western provinces is approximately 694 km, and they are 776 and 1101 km away from Ubon Ratchathani, respectively (**Fig 1**).

### Microsatellite genotyping

Ten MS markers (MS1, MS2, MS5, MS6, MS7, MS9, MS10, MS12, MS15 and MS20) previously used to differentiate *P*. *vivax* populations [18] were selected for genotyping (**S4 Table**). A nested PCR protocol was applied, with a 10-plex primary PCR followed by individual semi-nested PCRs for each marker, using a 40-fold dilution of the primary PCR product as the template [35]. The size of the PCR product was assessed by capillary electrophoresis in a 3730 XL ABI Sequencer (Applied Biosystems, Macrogen, South Korea). Electropherograms were analyzed using Peak Scanner v.2 (Macrogen, Seoul, South Korea). Only peaks with a height of ≥200 relative fluorescence unit were selected, and in case of several peaks only those with an intensity of at least one-third of the dominant peak.

### Data analysis

Isolates containing more than one peak for any marker were considered to be multiple clone infections. MOI of a given isolate was measured as the highest number of observed alleles at any of the 10 loci, or at any of the two most diverse loci. Alleles were binned using TANDEM software [36]. The mean number of alleles, allelic richness and expected heterozygosity (*H*_*E*_) were calculated using FSTAT v.2.9.3 [37]. Pairwise genetic differentiation (Weir & Cockerham *Fst* values) was calculated using FSTAT v.2.9.3. *Fst* varies from 0 (no genetic differentiation among populations) to 1 (no shared alleles among populations). LD was calculated using the program LIAN 3.7 [38] with 50,000 iterations for burn-in and then 100,000 Markov Chain Monte Carlo (MCMC) iterations. Samples with missing data were excluded for this analysis.

### Determination of an optimal set of MS markers for population differentiation

To evaluate the optimal panel of MS markers for differentiating between different clones in Thai parasite isolates, MS markers were removed in a stepwise manner [4]. The number of haplotypes after removing one marker at a time was counted using GenAlEx 6.5 [39] and expressed as the percentage of haplotypes identified compared to the full panel of 10 markers. The correlation between geographic and genetic distances was done by using Mantel rank test in GenAlEx 6.5.

### Population structure

STRUCTURE 2.3.2 software was used to assess clustering of haplotypes [40]. Twenty iterations were run for each cluster (K = 1–12) with a burn-in of 50,000 steps and then 500,000 MCMC steps using the admixture model. The cluster number K = 3 is considered optimal based on its consistency with other clustering methods including the principal component analysis and the phylogenetic tree analysis. PCoA was conducted by using the GenAlEx 6.5. The goeBURST algorithm within PHYLOViZ 2.0 was used to generate a unique minimum spanning tree [41]. Any haplotype with missing data was excluded from the tree. The unique tree was ruled by the n Locus Variants level (nLV) method, where n is the number of MS markers. All 116 complete multilocus haplotypes of the total 127 isolates were included in the tree.

### Effective population size (*Ne*)

The effective population size was calculated using two models, SMM and IAM, following the formula *Neμ* = 1/8{[1/1-*H*_*E*__mean]^2^–1 and *Neμ* = *H*_*E*__mean/4 (1-*H*_*E*__mean), respectively. *H*_*E*__mean is the average of the expected heterozygosity across all loci, while *μ* is the mutation rate per generation. Mutation rate values were derived from *P*. *falciparum*: 1.59 × 10^−4^ (95% confident interval 3.7 × 10^−4^–6.98 × 10^−5^) [42].

### Bottleneck analysis

Assuming the SMM [43], we assessed whether our results deviate from the mutation-drift equilibrium [44]. Alternative models exist, including the IAM and the Two-phase Model [45,46]. We chose the SMM because it is considered most appropriate for the interpretation of simple repeat units of microsatellite data [43].

## Supporting information

S1 Table The mean number of alleles (A) and allelic richness (B) of each microsatellite in the three populations.
(DOCX)

S2 Table Shared haplotypes based on genotyping using the 10 microsatellites (total number of haplotypes = 124). (DOCX)

S3 Table Remaining haplotypes (% and number) by the stepwise removal approach for all three populations (A), and individual populations from Ubon Ratchathani (B), Tak (C) and Kanchanaburi (D).
(DOCX)

S4 Table Primer sets for 10 microsatellite markers for the primary and semi-nested PCR. (DOCX)

S1 Fig Measurements of allelic numbers and allelic richness per locus. (TIFF)

S2 Fig Association between genetic and geographic distances by the Mantel Rank test. The correlation between genetic and geographic distance were examined by the Mantel rank test in GenAlEx 6.5. Analysis was done pairwise, using isolates within and between provinces. The X-axis represents the pairwise geographic distance and the Y-axis indicates corresponding genetic distance. Positive correlation was shown with *R*^2^ = 0.1993 at *p* < 0.05.
(TIF)
