# Supplementary material for: Substantial population structure of Plasmodium vivax in Thailand facilitates identification of the sources of residual transmission
Source: PLoS Negl Trop Dis. 2017 Oct 16;11(10):e0005930. doi: 10.1371/journal.pntd.0005930 (PMC5658191; doi:10.1371/journal.pntd.0005930)
Supplement: S1 Table — The mean number of alleles (A) and allelic richness (B) of each microsatellite in the three populations. (DOCX) [file pntd.0005930.s001.docx]

**S1 Table.** The number of alleles and allelic richness of each microsatellite in the three populations.

| Markers | Tak | | | Kanchanaburi | | | Ubon Ratchathani | | | All sites | | |  |
| --- | --- | --- | --- | --- | --- | --- | --- | --- | --- | --- | --- | --- | --- |
|  | No. alleles | *Shared alleles* | *Richness* | *No.*  *alleles* | *Shared alleles* | *Richness* | *No. alleles* | *Shared alleles* | *Richness* | *No. alleles* | *Shared alleles* | *Richness* | |
| MS1 | 6 | *4* | 5.673 | 7 | *5* | 6.967 | 6 | *5* | 6 | 13 | *2* | 9.421 | |
| MS2 | 18 | *11* | 16.700 | 18 | *11* | 17.901 | 17 | *12* | 16.997 | 40 | *0* | 28.617 | |
| MS5 | 13 | *11* | 12.708 | 9 | *6* | 9.000 | 10 | *8* | 9.999 | 16 | *4* | 13.460 | |
| MS6 | 17 | *12* | 16.017 | 12 | *6* | 11.934 | 9 | *5* | 8.998 | 38 | *0* | 26.578 | |
| MS7 | 15 | *10* | 14.066 | 13 | *6* | 14.066 | 11 | *6* | 11.000 | 34 | *0* | 24.091 | |
| MS9 | 12 | *5* | 10.891 | 13 | *8* | 12.969 | 14 | *8* | 14.000 | 28 | *0* | 20.962 | |
| MS10 | 13 | *6* | 11.803 | 10 | *5* | 9.969 | 8 | *6* | 7.999 | 24 | *0* | 16.938 | |
| MS12 | 16 | *12* | 15.275 | 7 | *5* | 6.988 | 9 | *5* | 8.998 | 31 | *0* | 23.821 | |
| MS15 | 11 | *6* | 10.104 | 5 | *4* | 4.994 | 7 | *5* | 6.999 | 22 | *0* | 17.113 | |
| MS20 | 16 | *9* | 14.824 | 12 | *7* | 11.975 | 11 | *7* | 10.998 | 38 | *0* | 27.552 | |
