# Supplementary material for: Substantial population structure of Plasmodium vivax in Thailand facilitates identification of the sources of residual transmission
Source: PLoS Negl Trop Dis. 2017 Oct 16;11(10):e0005930. doi: 10.1371/journal.pntd.0005930 (PMC5658191; doi:10.1371/journal.pntd.0005930)
Supplement: S2 Table — (DOCX) [file pntd.0005930.s002.docx]

**S2 Table.** Shared haplotypes based on genotyping using the 10 microsatellites (total number of haplotypes = 124).

| Haplotype | Count | Percent | Isolates | Site |
| --- | --- | --- | --- | --- |
| Hap25 | 2 | 1.61 | VTTY54 &59 | Tak |
| Hap28 | 2 | 1.61 | VKTS100 & VKBT114 | Kanchanaburi |
| Hap79 | 2 | 1.61 | VTTY12 & 41 | Tak |
| Hap92 | 4 | 3.22 | VTTY4, 16, 38, & 53 | Tak |
| Hap109 | 3 | 2.41 | VTTY6, 66 & 72 | Tak |

*There was no shared haplotype among the three provinces. Total of 111 haplotypes were unique.
