# Supplementary material for: Substantial population structure of Plasmodium vivax in Thailand facilitates identification of the sources of residual transmission
Source: PLoS Negl Trop Dis. 2017 Oct 16;11(10):e0005930. doi: 10.1371/journal.pntd.0005930 (PMC5658191; doi:10.1371/journal.pntd.0005930)
Supplement: S3 Table — Remaining haplotypes (% and number) by the stepwise removal approach for all three populations (A), and individual populations from Ubon Ratchathani (B), Tak (C) and Kanchanaburi (D). (DOCX) [file pntd.0005930.s003.docx]

**S3 Table.** Remaining haplotypes (% and number) by the stepwise removal approach for all three populations and individual populations from Ubon Ratchathani, Tak and Kanchanaburi.

| MS count | Residual haplotypes (%) | Haplotype count | Removed MS | Panel |
| --- | --- | --- | --- | --- |
| All Three populations | | | | |
| 10 MS | 100 | 116 |  | MS1, MS2, MS5, MS6, MS7, MS9, MS10, MS12, MS15 & MS20 |
| 9 MS | 100 | 116 | MS1 | MS2, MS5, MS6, MS7, MS9, MS10, MS12, MS15 & MS20 |
| 8 MS | 99.14 | 115 | MS5 | MS2, MS6, MS7, MS9, MS10, MS12, MS15 & MS20 |
| 7 MS | 98.28 | 114 | MS10 | MS2, MS6, MS7, MS9, MS12, MS15 & MS20 |
| 6 MS | 98.28 | 114 | MS15 | MS2, MS6, MS7, MS9, MS12 & MS20 |
| 5 MS | 97.41 | 113 | MS9 | MS2, MS6, MS7, MS12 & MS20 |
| 4 MS | 96.55 | 112 | MS7 | MS2, MS6, MS12 & MS20* |
| 3 MS | 94.83 | 110 | MS12 | MS2, MS6 & MS20 |
| 2 MS | 81.90 | 95 | MS6 | MS5 & MS2 |
| 1 MS | 33.62 | 39 | MS2 | MS5 |
| Ubon Ratchathani | | | | |
| 10 MS | 100 | 33 |  | MS1, MS2, MS5, MS6, MS7, MS9, MS10, MS12, MS15 & MS20 |
| 9 MS | 100 | 33 | MS10 | MS1, MS2, MS5, MS6, MS7, MS9, MS12, MS15 & MS20 |
| 8 MS | 100 | 33 | MS1 | MS2, MS5, MS6, MS7, MS9, MS12, MS15 & MS20 |
| 7 MS | 100 | 33 | MS15 | MS2, MS5, MS6, MS7, MS9, MS12 & MS20 |
| 6 MS | 100 | 33 | MS6 | MS2, MS5, MS7, MS9, MS12 & MS20 |
| 5 MS | 100 | 33 | MS12 | MS2, MS5, MS7, MS9 & MS20 |
| 4 MS | 100 | 33 | MS5 | MS2, MS7, MS9 & MS20* |
| 3 MS | 100 | 33 | MS7 | MS2, MS9 & MS20 |
| 2 MS | 96.97 | 32 | MS20 | MS2 & MS9 |
| 1 MS | 51.52 | 17 | MS9 | MS2 |
| Tak | | | | |
| 10 MS | 100 | 47 |  | MS1, MS2, MS5, MS6, MS7, MS9, MS10, MS12, MS15 & MS20 |
| 9 MS | 100 | 47 | MS9 | MS1, MS2, MS5, MS6, MS7, MS10, MS12, MS15 & MS20 |
| 8 MS | 100 | 47 | MS1 | MS2, MS5, MS6, MS7, MS10, MS12, MS15 & MS20 |
| 7 MS | 100 | 47 | MS15 | MS2, MS5, MS6, MS7, MS10, MS12, & MS20 |
| 6 MS | 97.87 | 46 | MS10 | MS2, MS5, MS6, MS7, MS12, & MS20 |
| 5 MS | 95.74 | 45 | MS7 | MS2, MS5, MS6, MS12, & MS20 |
| 4 MS | 95.74 | 45 | MS5 | MS2, MS6, MS12, & MS20* |
| 3 MS | 95.74 | 45 | MS20 | MS2, MS6 & MS12 |
| 2 MS | 85.11 | 40 | MS2 | MS6 & MS12 |
| 1 MS | 34.04 | 16 | MS6 | MS12 |
| Kanchanaburi | | | | |
| 10 MS | 100 | 36 |  | MS1, MS2, MS5, MS6, MS7, MS9, MS10, MS12, MS15 & MS20 |
| 9 MS | 100 | 36 | MS1 | MS2, MS5, MS6, MS7, MS9, MS10, MS12, MS15 & MS20 |
| 8 MS | 100 | 36 | MS15 | MS2, MS5, MS6, MS7, MS9, MS10, MS12 & MS20 |
| 7 MS | 100 | 36 | MS12 | MS2, MS5, MS6, MS7, MS9, MS10 & MS20 |
| 6 MS | 100 | 36 | MS10 | MS2, MS5, MS6, MS7, MS9 & MS20 |
| 5 MS | 100 | 36 | MS7 | MS2, MS5, MS6, MS9 & MS20 |
| 4 MS | 97.22 | 35 | MS5 | MS2, MS6, MS9 & MS20* |
| 3 MS | 91.67 | 33 | MS6 | MS2, MS9 & MS20 |
| 2 MS | 88.89 | 32 | MS20 | MS2 & MS9 |
| 1 MS | 50.00 | 18 | MS9 | MS2 |

*For each population or all populations together, the optimal subset of MS markers for further differentiation the respective population are highlighted.
