# Supplementary material for: Substantial population structure of Plasmodium vivax in Thailand facilitates identification of the sources of residual transmission
Source: PLoS Negl Trop Dis. 2017 Oct 16;11(10):e0005930. doi: 10.1371/journal.pntd.0005930 (PMC5658191; doi:10.1371/journal.pntd.0005930)
Supplement: S4 Table — (DOCX) [file pntd.0005930.s004.docx]

**S4 Table.** Primer sets for 10 microsatellite markers for the primary and semi-nested PCR.

| **Oligo name** | **Sequence (5’-3’)** | **5’Dye** |
| --- | --- | --- |
| MS1F | CATCTCGACATGTCGACGTAG | - |
| MS1R | ctgtcttTTGCTGCGTTTTTGTTTCTG | - |
| MS1nF | TCAACTGTTGGAAGGGCAAT | 6FAM |
| MS2F | AGCACGACCAACAAGAGAGG | - |
| MS2R | ctgtcttTGGGGAGAGACTCCCTTTTC | - |
| MS2nF | GAGCTAGCCAAAGGTTCAACA | 6FAM |
| MS5F | TTCGGCTGGTTTCCAATTAGG | - |
| MS5R | ctgtcttAAAGGGAGAGGAGCGAAAAC | - |
| MS5nF | CGTCCTCTATCGCGTACACA | NED |
| MS6F | GAGCTGCTGCTTCTATTTTGGG | - |
| MS6R | ctgtcttGGAGGACATCAACGGGATT | - |
| MS6nF | GGTTCTTCGGTGATCTCTGC | VIC |
| MS7F | ACATCAAAGCAAAGAAGAGGG | - |
| MS7R | ctgtcttAGGGTCTTCAGCGTGTTGTT | - |
| MS7nF | TTGCAGAAAATGCAGAGAGC | 6FAM |
| MS9F | TGAATTTCCCCATTTGCCCG | - |
| MS9R | ctgtcttGAAGCTGCCCATGTGGTAAT | - |
| MS9nF | AGATGCCTACACGTTGACGA | VIC |
| MS10F | AGGACCAAACGGAGGACATG | - |
| MS10R | ctgtcttTCCTTCAGGTGGGACTTGTT | - |
| MS10nF | TTATCCCTGCTGGATGTGAA | 6FAM |
| MS12F | AACGTTTCCTTGCCCACTTG | - |
| MS12R | ctgtcttCTGCTGTTGTTGTTGCTGCT | - |
| MS12nF | AATGCGCATCCTATGTCTCC | NED |
| MS15F | CGCACTCTTCATCCTCATCG | - |
| MS15R | ctgtcttCGGCCAGATGAAAAGGATAA | - |
| MS15nF | TGTTTGCAAAGGAATCCACA | VIC |
| MS20F | CAAGGTGCGATGGAAGATTGG | - |
| MS20nR | ctgtcttATTACTTTGTCGTAGTCCTCGGCGTAGTCC | - |
| MS20nF | GCACAACAAATGCAAGATCC | NED |
