# Supplementary figures and images for: Substantial population structure of Plasmodium vivax in Thailand facilitates identification of the sources of residual transmission
Source: PLoS Negl Trop Dis. 2017 Oct 16;11(10):e0005930. doi: 10.1371/journal.pntd.0005930 (PMC5658191; doi:10.1371/journal.pntd.0005930)

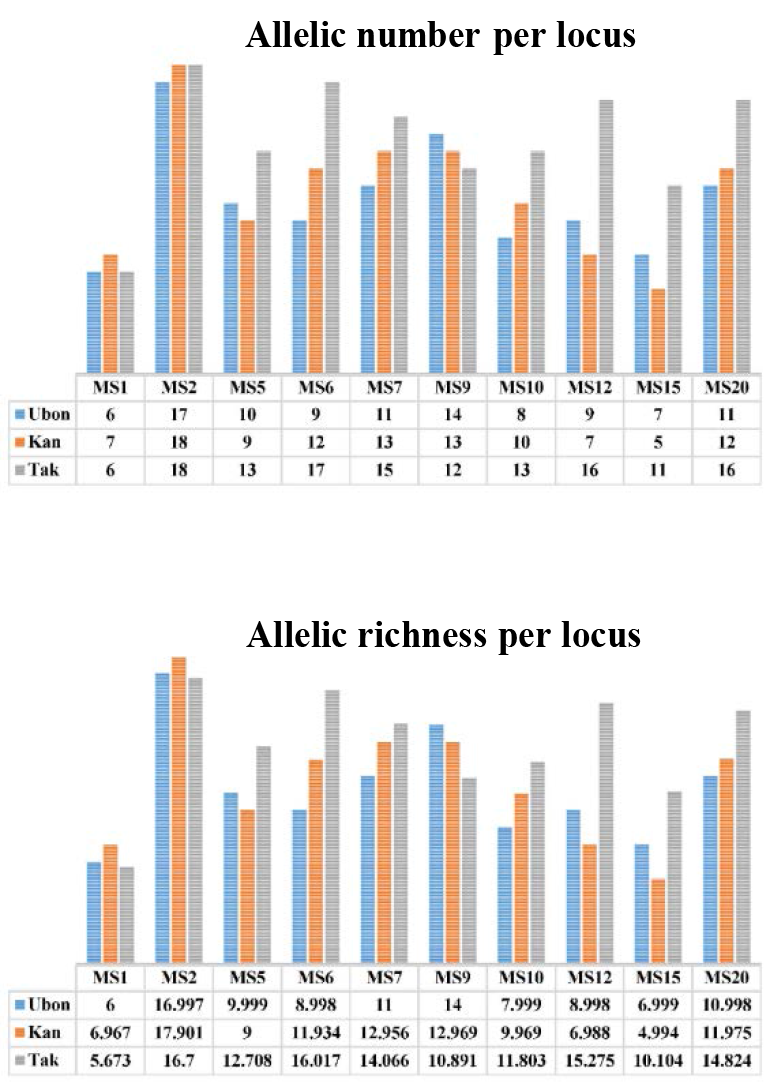

Supplement: S1 Fig — (TIFF) [file pntd.0005930.s005.tiff]

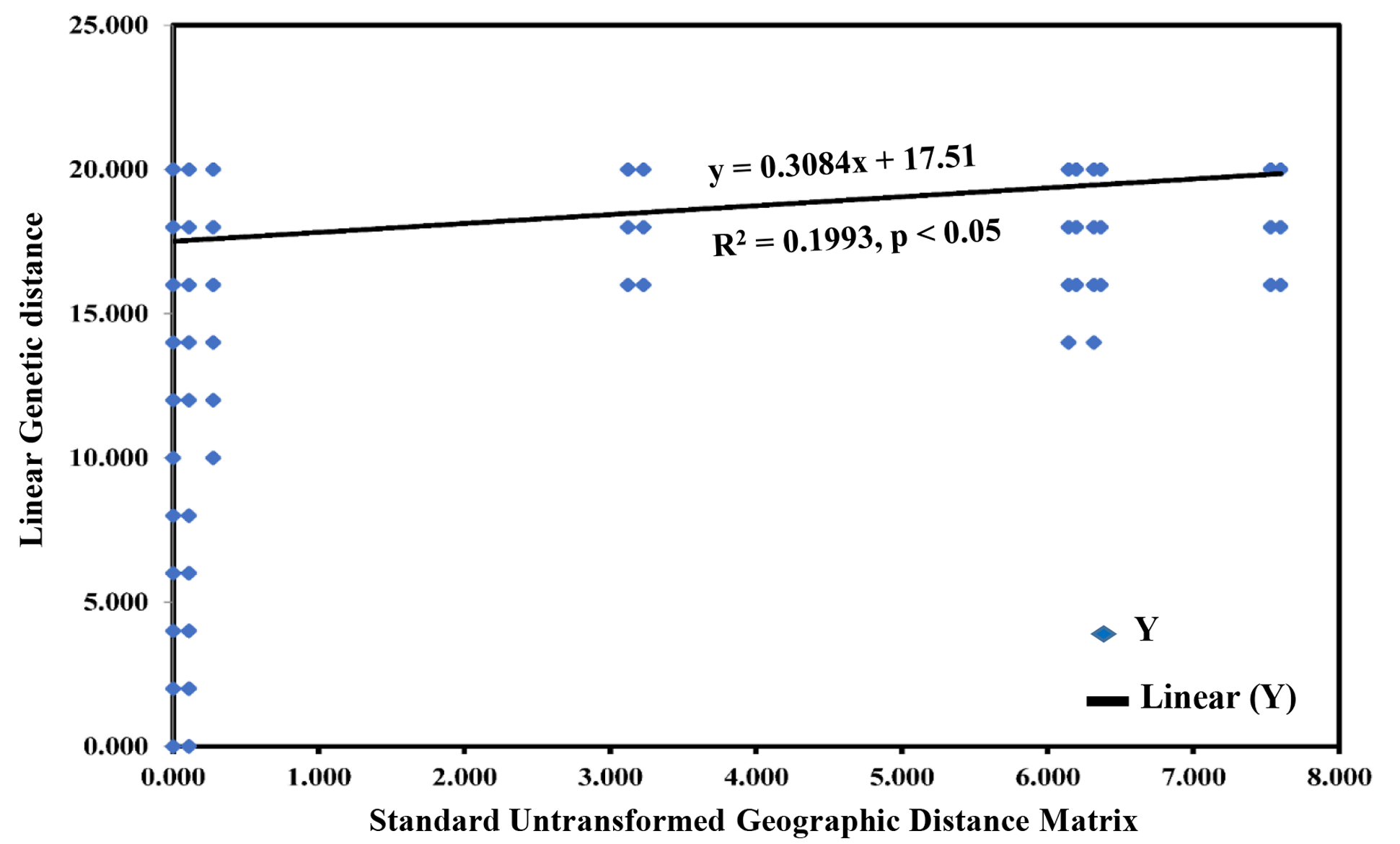

Supplement: S2 Fig — The correlation between genetic and geographic distance were examined by the Mantel rank test in GenAlEx 6.5. Analysis was done pairwise, using isolates within and between provinces. The X-axis represents the pairwise geographic distance and the Y-axis indicates corresponding genetic distance. Positive correlation was shown with R2 = 0.1993 at p < 0.05. (TIF) [file pntd.0005930.s006.tif]
